# Optimization of the Post-Process Heat Treatment Strategy for a Near-α Titanium Base Alloy Produced by Laser Powder Bed Fusion

**DOI:** 10.3390/ma15031032

**Published:** 2022-01-28

**Authors:** Christian Fleißner-Rieger, Tanja Pfeifer, Christoph Turk, Helmut Clemens

**Affiliations:** 1 Department of Materials Science, Montanuniversität Leoben, Franz-Josef Straße 18, 8700 Leoben, Austria; helmut.clemens@unileoben.ac.at; 2 Pankl Racing Systems AG, Additive Manufacturing Technologies, Industriestraße Ost 4, 8605 Kapfenberg, Austria; tanja.pfeifer@pankl.com; 3 Voestalpine BÖHLER Edelstahl GmbH & Co. KG, Mariazeller Straße 25, 8605 Kapfenberg, Austria; christoph.turk@bohler-edelstahl.at

**Keywords:** titanium alloys, additive manufacturing, characterization, electron microscopy, hardness, X-ray analysis, phase transformation

## Abstract

During the last decades, titanium alloys have been of great interest for lightweight applications due to their high strength in combination with a low material density. Current research activities focus on the investigation of near-α titanium alloys produced by laser powder bed fusion (LPBF). These alloys are known for their superior tensile strength and high creep resistance. This study focuses on the optimization of post-process heat treatments and the impact on tensile and creep strength of a LPBF produced Ti6242S alloy. Therefore, a variety of annealing steps were conducted to gain knowledge about the decomposition process of the non-equilibrium as-built microstructure and the arising influence on the mechanical properties. Components made of Ti6242S and produced by LPBF reveal an extraordinarily high ultimate tensile strength of about 1530 MPa at room temperature, but show a low elongation at fracture (A_5_ = 4.3%). Based on microstructure-property relationships, this study recommends precise heat treatments on how to improve the desired mechanical properties in terms of strength, ductility as well as creep resistance. Moreover, this study shows a triplex heat treatment, which enhances the elongation at fracture (A_5_) to 16.5%, while the ultimate tensile strength is still at 1100 MPa.

## 1. Introduction

Titanium base alloys are frequently used in the aerospace, automotive and medical sectors due to their beneficial strength-to-weight ratio, excellent corrosion resistance, biocompatibility, and high fatigue strength [1,2,3,4]. In particular, the group of the α + β alloys is of great interest because of their well-balanced properties and the already available know-how in terms of producibility [5,6,7]. Recent research activities have focused on the investigation of near-α titanium base alloys, which reveal similarities to the popular α + β alloys such as Ti-6Al-4V (m.%, Ti64), yet containing a lower fraction of β stabilizing elements [7,8,9,10]. Especially the near-α Ti-6Al-2Sn-4Zr-2Mo-Si (in m.%, Ti6242S) alloy produced by LPBF has gained attention due to several advantages such as higher tensile strength and superior creep resistance in comparison to the Ti64 alloy [11]. Additive manufacturing, e.g., LPBF, is regarded as a promising technology when compared to conventional manufacturing techniques, which is caused by less material waste via the direct production of highly complex geometries [12]. For example, titanium alloys are a popular choice for LPBF in industrial applications for producing biomedical implants [13], lightweight aerospace parts [14], as well as complex parts of the exhaust system in the automobile sector [11]. Choosing LPBF as a manufacturing technique can significantly improve the mechanical properties of components. Conventionally manufactured Ti6242S samples (casting and annealed) reveal an ultimate tensile strength (UTS) of 1000 MPa and an elongation at fracture of 10% [15]. The LPBF manufacturing process of Ti6242S components causes high cooling rates, leading to a martensitic microstructure and, in further consequence, to a significantly higher UTS of 1380 MPa and comparably low elongation at fracture of 5.3% [8]. The high cooling rates of this manufacturing process evoke a non-equilibrium material condition and promote a high density of lattice defects and residual stresses, which may lead to the formation of cracks and delamination during the LPBF process [16,17]. The microstructure of the Ti6242S components consists of long columnar parent β-grains (~83 µm width), several hundred µm length which contain fine acicular αʹ martensite (~1 µm minor axis length and ~8 µm major axis length) as recorded in [11]. 

The martensitic microstructure is far from thermodynamic equilibrium and, therefore, unfavorable for industrial application as it causes low ductility and fracture toughness. Therefore, post-process heat treatments are well established for LPBF-produced titanium alloys [8,18]. These treatments focus on the reduction in residual stresses and enhancement of ductility. Simultaneously, the heat-treated specimens’ tensile strength drops due to the change of a fully martensitic αʹ microstructure into an α + β microstructure which is closer to the thermodynamic equilibrium [19,20]. 

Fundamental investigations [21] on an LPBF-manufactured titanium base alloy show that the decomposition of the fully martensitic microstructure already begins at 400 °C. Annealing at this relatively low temperature increases the hardness in comparison to the initial sample condition. It is assumed that the presence of the intermetallic α_2_-Ti_3_Al phase and nanometer-sized β precipitates causes this hardness increase [16,22]. In contrast, heating to temperatures above 650 °C results in a higher β phase fraction as well as coarsened grains, leading to a hardness decrease [22]. After initial annealing, a final aging (stabilization) step is performed for elemental redistribution in the supersaturated phases, shifting the microstructure towards the thermodynamic equilibrium. For most Ti alloys, aging is typically performed in a temperature range of 500–600 °C, leading to a decomposition of unstable phases and a microstructure with enhanced hardness [23]. Furthermore, aging also promotes the formation of α_2_ precipitates, which are known to further strengthen the material by means of stabilizing the dislocation structure by dislocation pinning [23,24]. 

Recent investigations on an-LPBF manufactured Ti6242S alloy have shown that the heat treatment response led to the precipitation of β particles at α-grain boundaries and within α grains. This behavior was ascribed to the low diffusivity of the β-stabilizing element Mo in this alloy [11]. The Ti6242S alloy is also known for its high creep resistance caused by its low self-diffusion coefficient [5,7]. Moreover, the alloying element Si promotes the formation of stable (Ti,Zr)_6_Si_3_ silicide particles, which further decrease the dislocation movement [25]. 

This work aims to optimize the post-process heat treatment strategy of LPBF-manufactured Ti6242S samples with regard to balanced mechanical properties. Depending on the operating conditions, single or multi-step heat treatments are preferable. If the material is used at the upper-temperature limit (long term) at about 550 °C, then enhanced creep strength is required. In contrast, well-balanced ductility and strength are essential for room temperature (RT) application. Since the Ti6242S alloy was manufactured by LPBF for the first time only recently [8], appropriate annealing strategies have not yet been investigated in detail. Besides the impact of the heat treatment on the mechanical properties, additional focus is placed on the microstructural changes during the different steps of the post-process heat treatments. Particularly, the microstructure-property relationships concerning the tensile and creep strength will allow for identifying the proper heat-treatment strategy depending on the required mechanical properties. This may pave the way for a successful and more widespread use of the Ti6242S alloy produced by LPBF.

## 2. Materials and Methods

**Figure 1:**
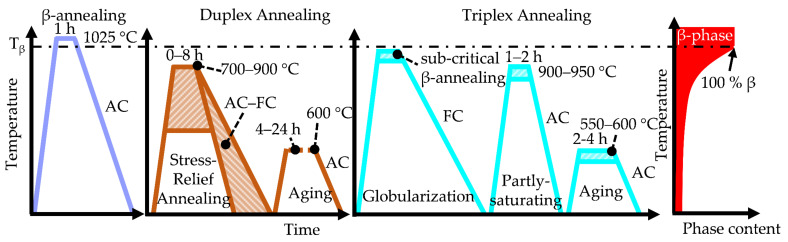
Schematic visualization of the investigated one-step, two-step and three-step post-process heat treatments conducted on as-built Ti6242S samples. The variation of temperatures, holding times and cooling conditions in terms of AC and FC was carried out to gain knowledge about the decomposition of the disequilibrium microstructure and to establish the optimum heat treatment parameters for the subsequent application. Additionally, schematically the course of the β phase fraction as function of temperature in thermodynamic equilibrium [11] is provided on the right side.

Microstructure analysis and mechanical testing were conducted on cylindrical specimens with a diameter of 15 mm and 85 mm length produced at Pankl Racing Systems AG, Additive Manufacturing Technologies, Kapfenberg, Austria on an M400 LPBF facility from EOS, Munich, Germany utilizing Ar shielding gas. The chosen volumetric energy density of the LPBF process was about 60 J mm^−3^ to ensure a high material density with a reduced pore content. The chemical analysis of the Ti6242S powder used for the manufacturing process was: 6.4 Al, 1.9 Sn, 3.9 Zr, 2.0 Mo, 0.09 Si, and 0.01 O (m.%). The chemical analysis of Al, Sn, Zr, Mo, Si was done via inductively coupled plasma atomic emission spectroscopy (ICP-AES), and the O concentration was determined via carrier gas hot extraction. Investigations on samples directly after the LPBF manufacturing process, i.e., on “as-built” samples, were conducted to analyze the reference condition and shed light on the starting microstructure. Dwell times, heat treatment temperatures and cooling conditions (air cooling, AC, furnace cooling, FC) were varied in order to learn about the microstructural evolution during the post-process treatments. Figure 1 displays the investigated one-step (β-annealing), two-step (duplex annealing) and three-step (triplex annealing) heat treatments investigated within this study. Based on thermodynamic calculations as reported in reference [11], a schematic diagram of the rising β phase fraction at higher temperatures is shown on the right of Figure 1.

The β phase fraction is of great importance due to the differences in solubility of chemical elements in the α and β phases [5]. Moderate annealing temperatures between 700 °C and 900 °C, which are significantly below the β-transus temperature (T_β_ for this alloy is 1017 °C, [11]), are commonly used in the industry to reduce residual stresses and enhance ductility [11]. For this purpose, this investigation focuses on the differences in mechanical properties caused by the varying temperatures and holding times during the duplex annealing treatment. A β-annealing treatment was executed to investigate the high temperature properties. Furthermore, a novel three-step triplex annealing treatment was designed to test the fracture at elongation limits of this alloy. All heat treatments were conducted in temperature-calibrated Nabertherm N 11/HR box furnaces. The sample preparation of the metallographic specimens was performed using standard preparation techniques for titanium alloys according to reference [26]. X-ray diffraction (XRD) investigations with a D8 Advance diffractometer from Bruker, Billerica, USA in coupled 2-θ mode using a Cu-K_α_ X-ray source with a wavelength of 0.154 nm were carried out to identify the occurring phases and the according volume fractions. Rietveld analysis [27] with the software TOPAS from Bruker was performed to analyze the phase fractions and lattice parameters of the phases present. Light optical microscopy (LOM) with an AxioImager.M2 LOM from Zeiss, Jena, Germany was performed to determine the pore content of the as-built samples according to reference [26]. Fractography was conducted on a stereo-microscope, type DiscoveryV20 from Zeiss. Scanning electron microscopy (SEM) was done on a field-emission device Versa 3D Dual Beam from Thermo Fisher, Waltham, USA. This SEM was also used to investigate the specimens’ phase morphology and grain size via electron backscatter diffraction (EBSD) analysis using a Hikari XP EBSD camera from EDAX, Mahwah, USA. The determination of the elemental composition of the individual phases was performed via energy-dispersive X-ray spectroscopy (EDS) on an Oxford Instruments Xmax 80 mm^2^ EDS detector, which was mounted on a Clara SEM from Tescan, Brno, Czech Republic. Transmission electron microscopy (TEM) investigations were carried out on an FEI Tecnai F20 G2 TEM from Thermo Fisher by using an acceleration voltage of 200 kV. Micro-hardness measurements were performed to examine the influence of the various heat treatment parameters on the specimens’ hardness. Therefore, a Qness Q 60 A+ measuring device from ATM Qness, Mammelzen, Germany equipped with a Vickers indenter tip was employed to perform 30 single HV0.1 indents per sample arranged on a measurement grid. Quasi-static tensile tests were conducted on heat-treated and machined tensile samples with gauge diameter of Ø 6 mm and a gauge length of 30 mm. The tensile specimens were mounted with a metric M10 screw head in accordance with DIN EN 2002 and deformed with a strain rate of 0.8· 10^−4^ s^−1^ on tensile test equipment provided by Messphysik. The listed elongation at fracture values A_5_ within this study were determined according to DIN EN 2002. All temperatures and sample conditions were tested at least two times. Uniaxial creep testing was carried at a temperature of 550 °C under an applied load of 210 MPa, using TC30 and TC50 creep testing equipment of AET Technologies, Troy, MI, USA. The cylindrical samples used for creep testing had an inner sample diameter of Ø 6 mm and a gauge length of 30 mm. During the experiment, two extensometer bars were attached to the specimens for creep strain determination. The temperature was monitored and controlled via three thermocouples along the specimens’ longitudinal axis. 

## 3. Results

### 3.1. As-Built Microstructure

**Figure 2:**
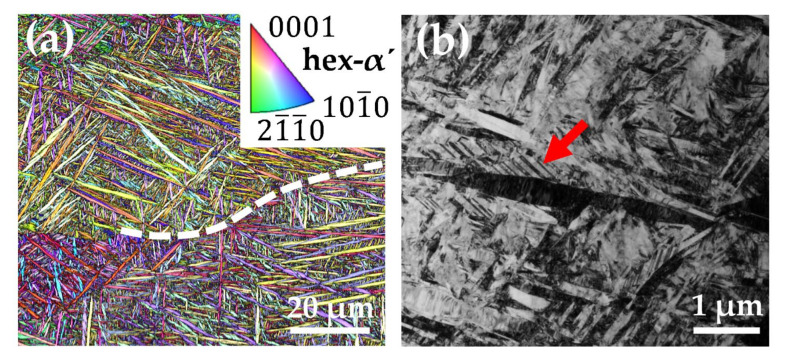
(**a**) EBSD IPF map with an IQ overlay shows the acicular morphology of the hexagonal martensitic microstructure after LPBF. A former parent β grain boundary is marked with a dashed line; (**b**) the TEM-BF image displays a high density of defects like mechanical twins (red arrow) in the nanometer-sized αʹ microstructure of the as-built sample.

The results of the electron microscopic investigations of an as-built sample are shown in Figure 2. Figure 2a displays the EBSD image quality (IQ) and inverse pole figure (IPF) map. The image reveals the acicular morphology of the as-built microstructure. As the material fulfills a β → αʹ phase transformation after solidification of the melt pool, grain boundaries of the parent β grains are still visible and marked with a dashed line. According to reference [11], the crystal orientation of the αʹ grains, located within the β grains, is linked to the Burgers orientation relationship. The pore analysis of the as-built samples reveals a very high density of the LPBF manufactured samples with a pore fraction of <0.05 vol.%.

Figure 2b shows a bright-field TEM (TEM-BF) image of the microstructure, which reveals a very fine αʹ microstructure and a high density of defects like mechanical twins (red arrow).

### 3.2. Transformation during Duplex Annealing

**Figure 3:**
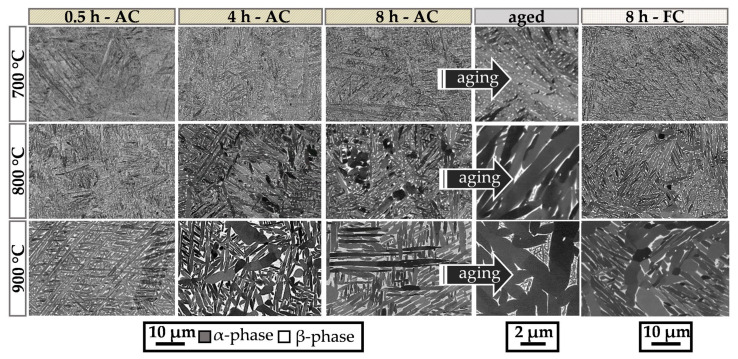
SEM images taken in BSE mode of the microstructure of LPBF-produced Ti6242S samples at different stages of the duplex annealing. Higher temperatures and longer dwell times lead to a significant increase in grain size. The additional aging treatment at 600 °C for 4 h results in a bi-lamellar morphology (see text).

Figure 3 shows SEM images of the microstructure recorded with a backscatter electron (BSE) detector to display the impact of different heat treatment temperatures and times on the evolution of the microstructure during the duplex annealing heat treatment. The images show the microstructure of the stress-relief annealed (SRA) samples at 700 °C, 800 °C and 900 °C from top to bottom and for dwell interval times of 0.5–8 h from left to right. Different cooling conditions in terms of AC and FC as well as a final aged microstructure are also added for comparison. As shown, the acicular morphology of the initial as-built sample condition remains for low annealing temperatures and short dwell times. Due to the decomposition of the supersaturated αʹ martensite (αʹ → α + β) closer to thermodynamic equilibrium, β precipitates have formed in all heat-treated specimens. The appearance of the β phase is bright in BSE contrast due to the enrichment with heavier elements like Mo [11]. An increase in the temperature to 900 °C and longer dwell times result in significant growth of α and β grains as well as a globularization of the whole microstructure.

In particular, the FC sample, which was annealed at 900 °C, reveals a much coarser microstructure originating from the low cooling rate. The β phase fraction in the FC samples is lower than in the AC condition, caused by the equilibrium condition of the material.

The effect of final aging (600 °C for 4 h) is visible when comparing the β phase before and after the aging treatment for the 8 h-AC sample in Figure 3. The aging step causes precipitation of fine α phase within former β areas. This so-called bi-lamellar microstructure originates as the supersaturated β phase shifts to thermodynamic equilibrium. The aging effect is evident, especially for high temperatures where large β phase areas exist. For lower annealing temperatures, e.g., 700 °C, the microstructure consists of sub-µm-sized β precipitates surrounded by α constituents. 

**Figure 4:**
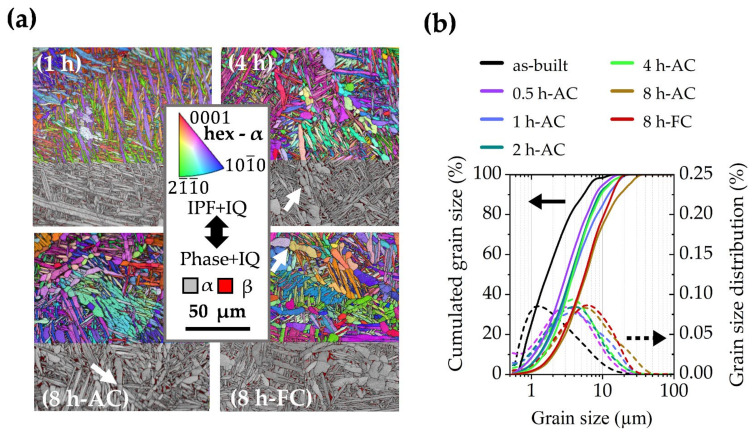
EBSD maps depicted as IPF + IQ maps on the upper side and as phase + IQ maps on the lower side of (**a**) showing the microstructure of the 900 °C heat-treated Ti6242S samples for different dwell times and cooling conditions. The EBSD grain size analysis in (**b**) reveals that the increasing grain size is linked to the prolonged dwell-time.

**Figure 5:**
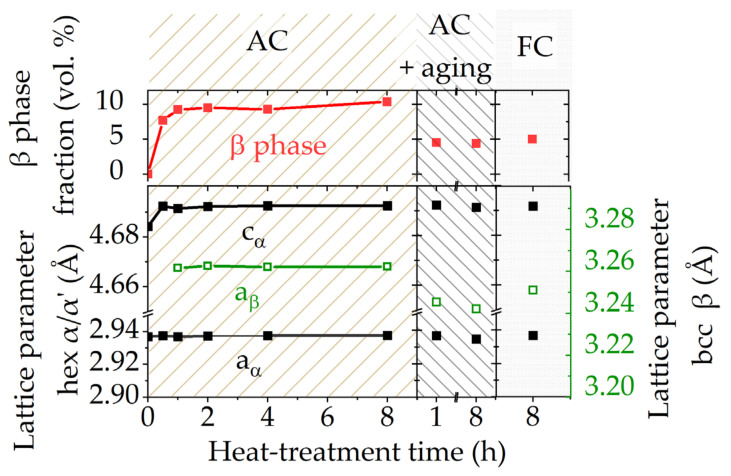
XRD results of the sample heat-treated at 900 °C showing the β phase volume fraction and lattice parameter of the α and β phase for different SRA dwell times and cooling conditions. The final aging step (600 °C for 24 h) was carried out on 900 °C-1 h-AC and 900 °C-8 h-AC samples.

EBSD investigations were performed to analyze the influence of the dwell time on the microstructure during the 900 °C annealing treatments (Figure 4). The upper areas in the EBSD maps in Figure 4a show the IPF + IQ information of the hex α phase, whereas the lower areas depict phase maps with an IQ overlay. The α phase is colorized in grey, whereas the β constituents are highlighted in red. The acicular appearance of the fine grains coarsens with increasing dwell times and larger α grains begin to split (white arrows). Furthermore, small β phase precipitates, as seen in the 1 h sample, accumulate and generate larger β grains in the 8 h treated samples. The EBSD grain size analysis in Figure 4b shows that an extension of the dwell time from 0.5 h to 8 h causes an increase in grain size. The average grain diameter d_50_ almost triples from 2 µm (as-built) to 5.9 µm (8 h-AC) due to the dissolution of smaller grains and the growth of the larger ones during the holding sequence. The evaluation of lattice parameters and phase fractions of the hexagonal (hex) α and body-centered cubic (bcc) β phase was done via XRD measurements and is depicted in Figure 5 for the samples heat-treated at 900 °C. The results of the AC samples reveal an increase in the β phase fraction already after a dwell time of 0.5 h and an almost steady volume fraction of about 10 vol.% after 1 h. The evaluation of the lattice parameters shows that the hex α lattice parameter (*a_α_* and *c_α_*) and the bcc β lattice parameter (*a_β_*) remain unaltered after the SRA-AC treatments. Final aging at 600 °C for 2 h was conducted on 1 h and 8 h SRAAC samples, which showed a decrease in the β phase fraction after the aging treatment as well as for lower cooling rates (FC), when compared to the AC treatment. A lower β phase fraction is also visible in the microstructure images of the samples heat-treated at 900 °C as shown in Figure 3.

**Figure 6:**
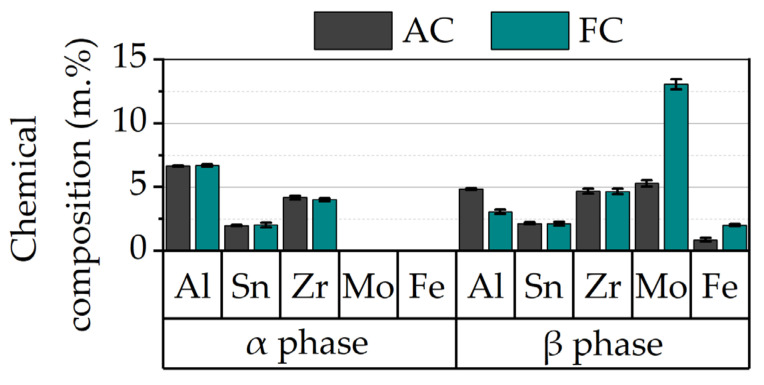
EDS measurements reveal the element partitioning between the α and β phase depending on the applied cooling rate.

Moreover, from Figure 5 it is evident that only the lattice parameter of the bcc β phase decreases during aging or FC. The change in the β lattice parameter results from differences in chemical composition. As shown in Figure 6, EDS measurements reveal nearly constant Al, Sn and Zr concentrations in the α phase, whereas the elements Mo and Fe completely diffuse into the β phase during the heat treatments. Obviously, the cooling rate severely influences the chemical composition of the β phase. While the FC sample is shifted closer to the thermodynamic equilibrium due to the lower cooling rate, the β phase is supersaturated in Al and depleted in Mo and Fe after the AC treatment. This is accompanied by a lower β phase fraction obtained in the specimens closer to equilibrium. The XRD diffractograms also suggest that no intermetallic α_2_-Ti_3_Al phase occurs in any of the investigated samples.

### 3.3. β-Annealing and Triplex Annealing

**Figure 7:**
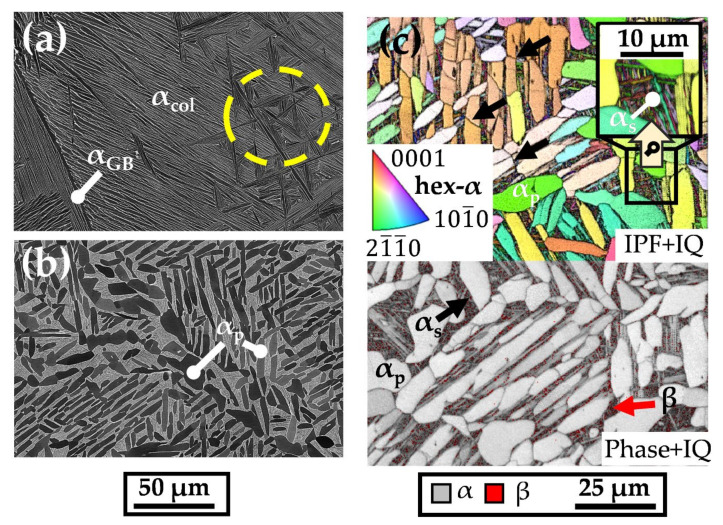
(**a**) SEM-BSE image shows the microstructure after β-annealing of an as-built Ti6242S sample. (**b**,**c**) demonstrate the effect of the triplex annealing heat treatment on the microstructure displayed in an SEM-BSE image and an EBSD map, respectively.

As a lamellar microstructure results in improved creep resistance of Ti base alloys [5], a β-annealing treatment above the β transus temperature was utilized to diminish the as-built morphology and generate a more coarse-grained and lamellar microstructure. The impact of this treatment on the microstructure is shown in Figure 7a. The microstructure consists of large primary α colonies (α_col_), grain boundary α (α_GB_) and a slight amount of basketweave substructures (encircled yellow) within a single parent β grain. 

Triplex annealing was performed to enhance the ductility of the as-built condition. An SEM-BSE image of the microstructure after the triplex annealing is depicted in Figure 7b. It consists of darkly contrasted, partially globularized primary α (α_p_) grains and coarse α_p_ lamellas which formed during the first stage of the three-step heat treatment, i.e., sub-critical annealing slightly below β-transus. In the second stage, the saturation annealing leads to approximately 30 vol.% of supersaturated β phase caused by the rapid cooling process. The black arrows indicate the splitting of α_p_ grains, which originated during the first two annealing steps, better visible in the EBSD map shown in Figure 7c. In the third step of the triplex annealing, aging promotes secondary α (α_s_) and fine β precipitation. Here, the IPF + IQ map on the upper side shows these small α_s_ constituents in between the α_p_ grains. In the enlarged box, the grain size analysis reveals an α grain size of about 1.0 µm when compared to the overall grain size of the triplex annealed sample of about 13.6 µm. Regarding the crystallographic orientation, α_p_ and α_s_ are both linked to the parent β phase via the Burgers orientation relationship [11]. Moreover, the EBSD Phase + IQ map on the lower side of Figure 7c presents the β phase (red arrow) occurring in the vicinity of α_s_ grain boundaries.

### 3.4. Impact of the Heat Treatments on the Mechanical Properties

**Figure 8:**
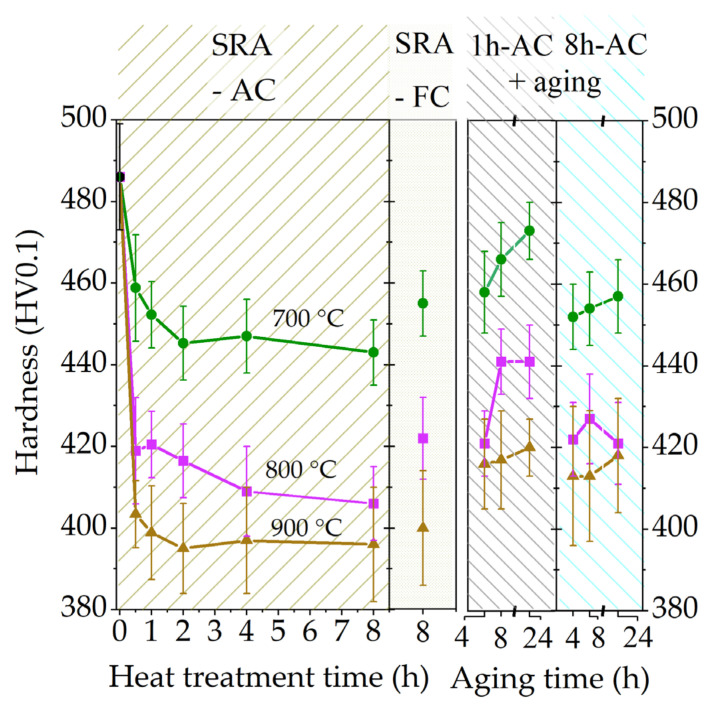
Micro-hardness tests performed on heat-treated Ti6242S specimens show a decreasing hardness with increasing heat treatment temperatures and times (**left**). Low cooling rates, such as FC and final aging at 600 °C, promote the hardness (**right**).

The results of the micro-hardness tests of the duplex-annealed Ti6242S samples are displayed in Figure 8. The hardness of all AC samples decreases as both heat treatment temperature and time increase. The high hardness value of the as-built condition (486 HV0.1) significantly drops in the early stages of the heat treatment, which is more pronounced for the samples annealed at temperatures of 800 °C and 900 °C when compared to the 700 °C samples. In addition, the steady-state hardness is reached after a dwell time of 2 h for the investigated SRA temperatures in the range of 700–900 °C. The effect of the final aging treatment (600 °C) during the last step of the duplex annealing is visible in the hardness depicted on the right of Figure 8. Aging was done on 1 h-AC (short SRA dwell time) and 8 h-AC (long SRA dwell time) samples and reveals an increase in micro-hardness, which is more pronounced for shorter SRA dwell times. It can be assumed that longer SRA dwell times lead to a more stable phase composition and, therefore, less pronounced hardening during aging. Higher aging times (4 h → 8 h **→** 24 h) are also more effective for shorter SRA dwell times, whereas longer dwell times are not as favorable with regard to the obtained micro-hardness. Lower cooling rates, such as FC, result in an increase in hardness, especially for lower SRA temperatures. During the FC process, the microstructure shifts towards thermodynamic equilibrium when compared to AC, and the β phase fraction, which is lower after FC, is enriched with β stabilizing elements. Furthermore, the increased α phase fraction also contributes to a higher micro-hardness after the FC process as, in general, the α phase possesses higher strength than the β phase [28,29]. FC might also have a similar effect on the microstructure, especially at lower SRA temperatures, as seen for the aging treatment which leads to sub-µm sized β precipitates.

**Figure 9:**
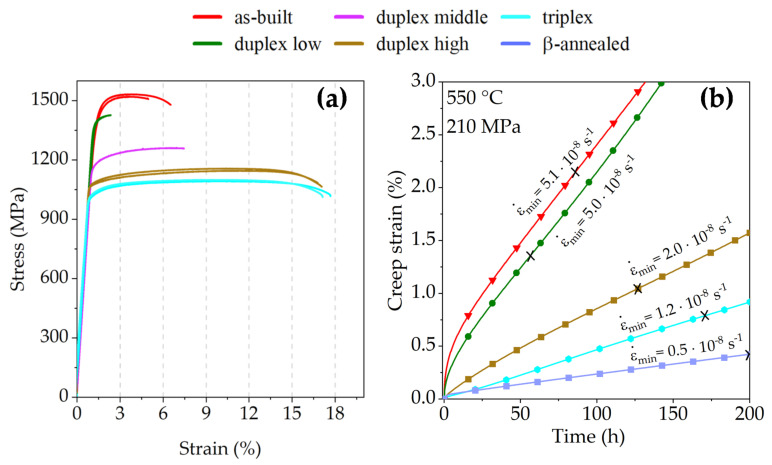
(**a**) Stress-strain curves obtained at RT show the tensile mechanical properties of the LPBF-produced Ti6242S specimens in as-built and heat-treated sample conditions; (**b**) creep strain as a function of time, which reveals the creep resistance as a function of the applied heat treatment. The cross (×) marks the time when the minimum creep rate (${\dot{\varepsilon }}_{\min}$) was reached.

Tensile testing was conducted to validate the performance of samples with selected heat treatments, having a special focus on the elongation at fracture. The results of the tensile tests are compiled in Figure 9a and Table 1. They confirm that the UTS is highest for the as-built sample condition of around 1525 MPa. During the low-temperature duplex annealing (“duplex low” = 700 °C + aging), the strength and, interestingly, the strain to rapture decreases when compared to the as-built samples. Fractography analysis on the duplex low samples reveals crack initiation sites at LPBF typical defects, such as pores. As shown in the microstructure images, these duplex-annealed samples consist of a very fine α + β microstructure with sub-µm-sized β precipitates and, thus, are severely influenced by the possible movement and pile-ups of dislocations [8]. Therefore, stress concentrations at defects lead to cracks and cause early fracture events compared to the “duplex high” samples (=900 °C + aging). These samples show an elongation at break (A_5_) of about 16.0%, although the UTS is still at 1155 MPa. The α + β microstructure within these samples can store continuously formed dislocations and offers sufficient space for dislocation movement in large α_p_ grains leading to enhanced ductility. The triplex heat treatment leads to a slight improvement of the ductility to 16.5 ± 0.3% (A_5_), caused by a microstructure almost composed of large α_p_ grains. 

Besides the tensile properties, additional focus is placed on the impact of the heat treatment on the creep resistance of the LPBF-manufactured Ti6242S components. Therefore, creep tests were conducted at 550 °C under an applied load of 210 MPa on all variants. Figure 9b displays the creep strain as a function of the time as well as the minimum creep rate values (${\dot{\varepsilon }}_{\min}$). The cross marks the time when the sample reached the minimum creep rate, also provided in Table 1. The as-built sample, which exhibits the highest tensile strength, reaches a creep strain ε > 1% already after 26 h and reveals a ${\dot{\varepsilon }}_{\min}$ of 5.1·10^−8^ s^−1^ after 85 h. The minimum creep rate decreases for the duplex annealed samples with increasing annealing temperatures from ${\dot{\varepsilon }}_{\min}$5.0·10^−8^ s^−1^ (duplex low) to ${\dot{\varepsilon }}_{\min}$2.0·10^−8^ s^−1^ (duplex high). The respective ε > 1% creep strain is reached after 37 h and 120 h under the applied load of 210 MPa. The triplex annealed sample shows a further improvement of creep resistance and a ${\dot{\varepsilon }}_{\min}$ min of 1.2· 10^−8^ s^−1^. For this sample condition, a creep strain of ε > 1% is only exceeded after 218 h. During β-annealing, the very fine α΄ martensite transforms into a lamellar α + β microstructure which is favorable for high creep resistance. This sample condition reveals the highest creep resistance, where ${\dot{\varepsilon }}_{\min}$ of 0.5· 10^−8^ s^−1^ is reached after 200 h, and the ε > 1% creep strain was not reached until the end of the creep test, in this case after 350 h.

## 4. Discussion

The combination of the beneficial properties of Ti base alloys with the LPBF manufacturing process allows the production of light-weight structural parts with highly complex geometries. However, LPBF requires comprehensive know-how in the field of processing and in-depth knowledge of material-specific characteristics. Parts produced by LPBF reveal a non-equilibrium martensitic α΄ microstructure, including a high amount of lattice defects and residual stresses [11]. Therefore, sub-transus post-process heat treatments are an effective way to shift the microstructure and the chemical composition of the constituting phases towards thermodynamic equilibrium, i.e., αʹ → α + β. Due to the relationship of mechanical properties and microstructural features, the applied heat treatments must be chosen carefully and should be explicitly adapted to the requirements for the final application. Concerning the wide range of available heat treatments for Ti base alloys, the following findings provide a guide for setting up a tailor-made heat treatment strategy:

### 4.1. Heat Treatment to Achieve Higher Tensile Strength

In order to adjust a high tensile strength in a Ti6242S alloy, the as-built condition or duplex annealing is recommended. The tensile tests of the as-built samples show extraordinarily high tensile strength values of UTS = 1526 MPa, although the elongation at fracture A_5_ is still at 4.3%, see Table 1. Therefore, the use of as-built samples should be considered if applications primarily focus on strength and low operating temperatures. It should be noted that as-built samples additionally reveal melt pool borders, which occur normal to the building direction and are caused by element segregations during the LPBF process. These melt pool borders, however, are of minor importance for mechanical properties as the total difference of element concentrations is comparably low [11].

Duplex annealing is recommended for applications with a need for well-balanced properties in terms of fracture at elongation and strength. During SRA, the overall decreasing hardness is determined by an interaction of decreasing defect density, grain coarsening and fine β phase precipitation. Additional aging is essential to increase the strength via element partitioning and precipitation of secondary α features [23,24,30]. It is noteworthy that aging is only possible if the α and β phases are not stable and under- or supersaturated in alloying elements. After AC, the β phase is supersaturated in Al and depleted in Mo and Fe and tends to shift towards thermodynamic equilibrium during aging. This leads to the precipitation of secondary α_s_ and to a decrease in the β phase fraction. As a result, the remaining β phase is heavily stabilized and a solid solution hardening with Mo and Fe is expected [5].

Regarding the SRA temperatures, duplex annealing at 700 °C causes sub-µm-sized β-precipitates in combination with very fine α constituents. This results in high strength but significantly decreases the ductility due to the lack of large plastic zones and dislocation pile-ups. Therefore, higher SRA temperatures, such as 800 °C and 900 °C, are favorable. The extension of SRA dwell times from 2 to 8 h does not affect the hardness, yet is important if an additional aging step is conducted. While shorter SRA dwell times lead to an increase in hardness during aging from 4 h to 24 h for the “duplex low” (700 °C) and “duplex middle” (800 °C) samples, the “duplex high” (900 °C) sample is not affected by longer SRA and aging dwell times. To summarize, the duplex annealing should either be performed with short dwell times for “duplex middle” treatment to the benefit of the strength enhancement during aging (800 °C-1 h-AC–600 °C-24 h-AC) or with a longer dwell time for “duplex high” heat treatment to gain additional ductility (900 °C-8 h-AC–600 °C-4 h-AC). The hardness of FC samples, in contrast, cannot further be enhanced by aging as these samples are already in thermodynamic equilibrium. 

### 4.2. Triplex Annealing to Obtain High Ductility

In general, a fine grained equiaxed microstructure is beneficial for higher ductility and can be achieved by thermo-mechanical processing (TMP) [5]. As TMP is not possible for AM components, a proper heat treatment strategy must be developed. Triplex annealing consists of a globularization treatment slight below β-transus followed by FC. During this stage, the globularization of primary α_p_ grains occurs due to grain growth and so-called grain segmentation. The segmentation of primary grains results from the formation of subgrain boundaries caused by the minimization of the total free energy and the rearrangement of tangled dislocations into dislocation arrays as described in reference [31]. The large globularized α_p_ grains contribute to the ductility and reveal enhanced local plasticity as they are capable of containing a large number of slip and shear bands and elongate under external load. The follow-up annealing between 900 and 950 °C generates approximately 30 vol.% of supersaturated β phase. This phase shift towards thermodynamic equilibrium during the final aging step and generates very fine secondary α_s_, contributing to the strength by hindering the dislocation motion via generation dislocation pile-ups at the α/β phase boundaries. In addition, slip bands and dislocation walls in primary α_p_ also enhance the strength by providing an effective barrier against dislocation movement [31]. 

In this context, it should be mentioned that also proper duplex annealing leads to enhanced fracture at elongation during tensile testing. In addition, duplex annealing would not need a third annealing step when compared to triplex annealing. On the downside, however, increasing dwell times at high temperatures must be considered as they are essential for superior ductility after duplex annealing.

### 4.3. Enhanced Creep Resistance by Means of β Annealing

The Ti6242S alloy is known for a superior temperature resistance when compared to the Ti64 alloy, especially at temperatures above 500 °C [15]. To enhance the creep resistance in the Ti6242 alloy, a minor amount of Si is added, which results in the precipitation of (Ti,Zr)_6_Si_3_ silicide particles which decelerate the dislocation motion substantially [7,25,32]. According to previous investigations on a related alloy [33], creep in this alloy is governed by lattice diffusion-controlled dislocation climb. The coarse lamellar microstructure is beneficial for use at elevated temperatures [5], which was also confirmed in the creep investigations within this study. Therefore, this treatment should be used if applications primarily focus on temperature resistance. Unfortunately, the β-annealing treatment has to be performed above the β-transus temperature (T_β_ = 1017 °C). However, these high temperatures diminish the typical LPBF microstructure and, in further consequence, decrease the superior tensile properties resulting from the LPBF process. Moreover, the newly formed α phase at grain boundaries influences the mechanical properties negatively [5].

It is noteworthy that triplex annealed samples also show enhanced creep properties. With regard to the excellent creep resistance of the triplex annealed material, in combination with the well-balanced mechanical properties in terms of strength and ductility, this heat treatment strategy might also be applicable for the multifunctional usage of LPBF-manufactured Ti6242S parts.

## 5. Conclusions

This study sheds light on the process-microstructure-property relationship of an LPBF-manufactured Ti6242S alloy and provides recommendations for specific annealing strategies. The mechanical testing, in combination with a detailed microstructure investigation, utilizing a variety of high-resolution characterization techniques, led to the following conclusions:

- For high strength applications, it is recommended to use: (i) the as-built or (ii) the duplex annealing heat treatment (well balanced ductility-strength ratio).
- Engineering applications that require high ductility can be fulfilled if triplex or duplex annealing is performed. These treatments lead to a significant increase in the elongation at fracture.
- Annealing above the β-transus temperature T_β_ is recommended for long-term operating temperatures above 500 °C.

## Figures and Tables

**Table 1:** Comparison of mechanical properties and the influence of heat treatments on LPBF manufactured Ti6242S material.

Sample	Heat Treatment	UTS (MPa)	YS (MPa)	A_5_ (%)	Min. Creep Rate ${\dot{\varepsilon }}_{\min}$ (10^−8^ s^−1^)	Time (h) ε > 1%	Reference
as-built	-	1526 ± 6	1406 ± 17	4.3 ± 0.8	5.1 (85 h)	26	this study
duplex low	700 °C–1 h–AC 600 °C–24 h–AC	1421 ± 5	1390 ± 2	0.9 ± 0.3	5.0 (56 h)	37	this study
duplex middle	800 °C–1 h–AC 600 °C–24 h–AC	1263	1172	6.5	-	-	this study
duplex high	900 °C–8 h–AC 600 °C–4 h–AC	1155 ± 7	1075 ± 4	16.0 ± 0.1	2.0 (127 h)	120	this study
triplex	sub-critical β-annealing–FC 900–950 °C–1–2 h–AC 550–600 °C–2–4 h–AC	1098 ± 4	1018 ± 5	16.5 ± 0.3	1.2 (170 h)	218	this study
β-annealed	1025 °C–1 h–AC	-	-	-	0.5 (200 h)	>350	this study
as-built	-	1381	1293	5.3	-	-	[8]
cast + annealed	-	1006	910	10	-	-	[15]

## Data Availability

The datasets generated during and/or analyzed during the current study are available from the corresponding author on reasonable request.
